# Liver-expressed antimicrobial peptide 2 elevation contributes to age-associated cognitive decline

**DOI:** 10.1172/jci.insight.166175

**Published:** 2023-05-22

**Authors:** Jing Tian, Lan Guo, Tienju Wang, Kun Jia, Russell H. Swerdlow, Jeffrey M. Zigman, Heng Du

**Affiliations:** 1 Department of Pharmacology and Toxicology and; 2 Higuchi Biosciences Center, University of Kansas, Lawrence, Kansas, USA.; 3 Alzheimer’s Disease Center, University of Kansas Medical Center, Kansas City, Kansas, USA.; 4 Departments of Internal Medicine and Psychiatry, University of Texas Southwestern Medical Center, Dallas, Texas, USA.

**Keywords:** Aging, Neuroscience, Alzheimer disease, G protein&ndash;coupled receptors, Memory

## Abstract

Elderly individuals frequently report cognitive decline, while various studies indicate hippocampal functional declines with advancing age. Hippocampal function is influenced by ghrelin through hippocampus-expressed growth hormone secretagogue receptor (GHSR). Liver-expressed antimicrobial peptide 2 (LEAP2) is an endogenous GHSR antagonist that attenuates ghrelin signaling. Here, we measured plasma ghrelin and LEAP2 levels in a cohort of cognitively normal individuals older than 60 and found that LEAP2 increased with age while ghrelin (also referred to in literature as “acyl-ghrelin”) marginally declined. In this cohort, plasma LEAP2/ghrelin molar ratios were inversely associated with Mini-Mental State Examination scores. Studies in mice showed an age-dependent inverse relationship between plasma LEAP2/ghrelin molar ratio and hippocampal lesions. In aged mice, restoration of the LEAP2/ghrelin balance to youth-associated levels with lentiviral shRNA *Leap2* downregulation improved cognitive performance and mitigated various age-related hippocampal deficiencies such as CA1 region synaptic loss, declines in neurogenesis, and neuroinflammation. Our data collectively suggest that LEAP2/ghrelin molar ratio elevation may adversely affect hippocampal function and, consequently, cognitive performance; thus, it may serve as a biomarker of age-related cognitive decline. Moreover, targeting LEAP2 and ghrelin in a manner that lowers the plasma LEAP2/ghrelin molar ratio could benefit cognitive performance in elderly individuals for rejuvenation of memory.

## Introduction

Gradual deterioration of the hippocampus contributes to age-associated cognitive decline (1–3). The unsettling of memories accompanying normal aging, though subtle, affects the daily life of the elderly population, precipitating age-related cognitive disorders such as Alzheimer’s disease (AD) (4). So far, the mechanistic pathways that render hippocampal vulnerability to aging are poorly understood, and effective therapies that reverse age-related memory loss are unavailable. Ghrelin (also referred to in literature as “acyl-ghrelin”) is an octanoylated peptide hormone that signals through its receptor, growth hormone secretagogue receptor (GHSR), to induce a variety of effects on energy metabolism and cell functions in multiple organs and tissues (5). Consistent with the abundant expression of GHSR in hippocampal neurons (6–13) and the capability of circulating ghrelin to penetrate into deep brain regions, including the hippocampus (14, 15), ghrelin is emerging as a critical neurotropic peptide that regulates hippocampal synaptic function and neurogenesis, contributing to memory formation and consolidation as well as mood (13, 16–21). While previous studies have not reached a consensus regarding the age-related changes in ghrelin production and activation (22–24), ghrelin incapacitation that is independent of metabolic status has been reported in the elderly (25, 26). Indeed, several ghrelin mimetics have been investigated over the years as a treatment for frailty in older adults (26–28). In view of a close association of ghrelin signaling with cognition (13, 16–18), it would be of interest to determine the molecular mechanisms of ghrelin signaling deregulation during normal aging as well as the impact of this deregulation on the development of age-associated cognitive decline.

Liver-expressed antimicrobial peptide 2 (LEAP2) is a recently identified endogenous GHSR antagonist/inverse agonist that reduces both ghrelin-mediated and ghrelin-independent GHSR activity (29–32). Previous work has highlighted the effects of balanced and imbalanced LEAP2 and ghrelin levels on energy homeostasis. These works include studies that have investigated changes in the plasma LEAP2/ghrelin molar ratio in relation to body mass, feeding, and blood glucose; studies that have uncovered effects of administered LEAP2 or LEAP2 peptide fragments to reduce food intake and blood glucose; and studies that have demonstrated effects of neutralizing antibody– and shRNA-mediated LEAP2 knockdown and genetic *Leap2* deletion on growth hormone (GH) secretion, food intake, body weight, and hepatic fat accumulation (32–36). However, the status of plasma LEAP2 and the plasma LEAP2/ghrelin molar ratio in normal aging remains insufficiently studied. Here, we show an age-related elevation in circulating LEAP2 alongside a marginal decrease in ghrelin, resulting in an increased LEAP2/ghrelin molar ratio in a cohort of elderly participants without dementia. Within that elderly cohort, the LEAP2/ghrelin molar ratio was negatively correlated with degree of cognitive function. Furthermore, this observed age-related LEAP2/ghrelin molar ratio imbalance was recapitulated in aging mice and coincided with the hippocampal synaptic injury that is seen in younger GHSR-null mice. Redressing the LEAP2/ghrelin molar ratio by suppressing LEAP2 production improved cognition and mitigated hippocampal pathology in aged mice. Our results suggest that LEAP2 elevation accompanies aging and further imply that the imbalanced plasma LEAP2/ghrelin levels are related to the development of cognitive deficits with advanced age. Therefore, LEAP2 constitutes a promising therapeutic target for memory rehabilitation in the elderly.

## Results

### LEAP2/ghrelin imbalance is an age-related change in elderly participants without dementia.

**Figure 1:**
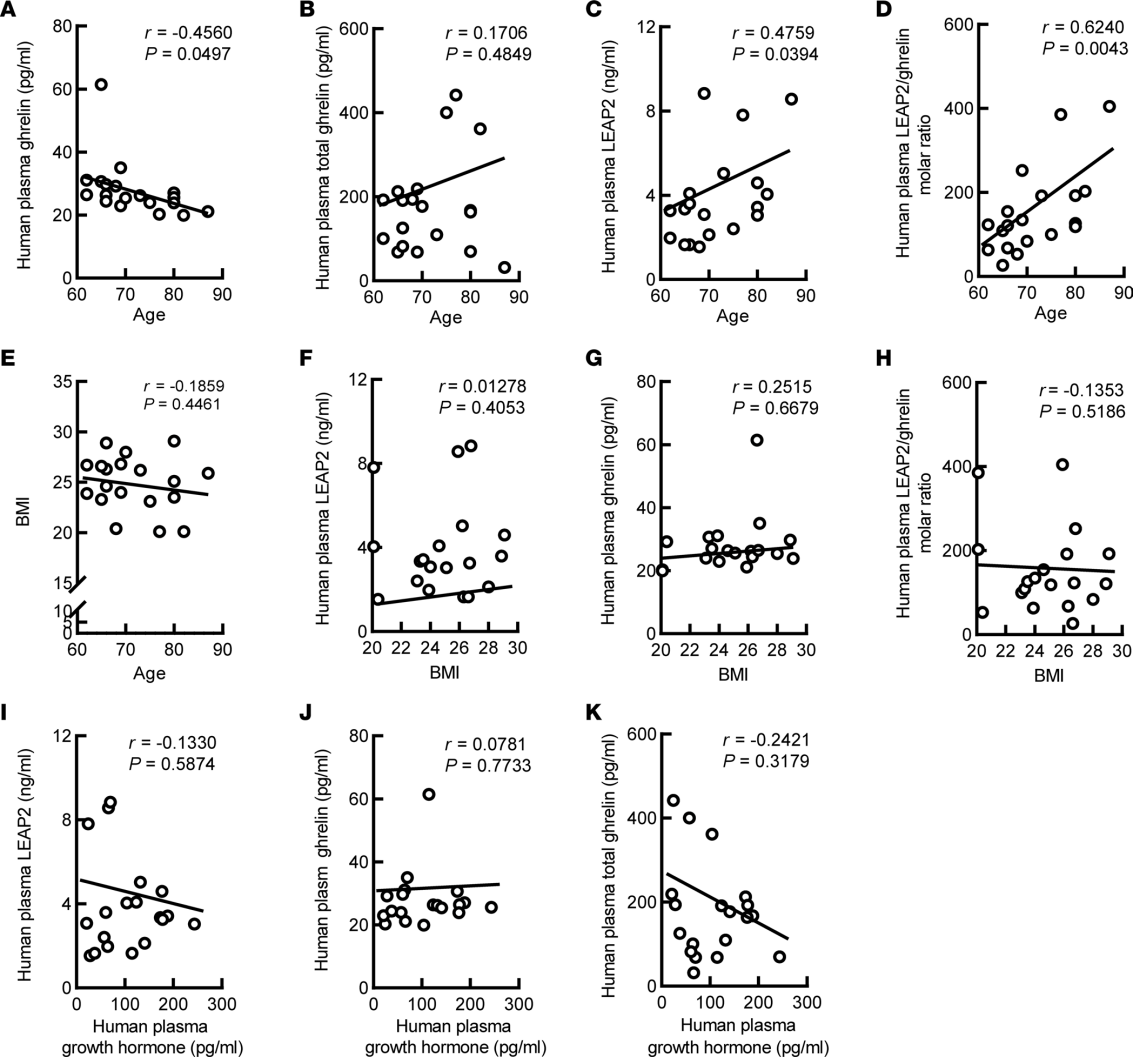
Age-associated change of plasma LEAP2, ghrelin, and LEAP2/ghrelin ratio in the elderly. (**A**–**E**) Correlation between age and plasma ghrelin (**A**), plasma total ghrelin (**B**), plasma LEAP2 (**C**), plasma LEAP2/ghrelin molar ratio (**D**), and BMI (**E**) were calculated using 2-tailed Pearson correlation coefficients. *n* = 19. (**F**–**H**) Correlation between BMI and plasma LEAP2 (**F**), plasma ghrelin (**G**), and plasma LEAP2/ghrelin molar ratio (**H**) were calculated using 2-tailed Pearson correlation coefficients. *n* = 19. (**I**–**K**) Correlation between plasma growth hormone and plasma LEAP2 (**I**), plasma ghrelin (**J**), and plasma total ghrelin (**K**) were calculated using 2-tailed Pearson correlation coefficients. *n* = 19.

To determine the status of circulating ghrelin and LEAP2 in aging, we performed ELISA for circulating LEAP2, ghrelin, and total ghrelin (that is, both ghrelin and des-acyl ghrelin, the latter of which does not bind GHSR and yet possesses some physiological activities that are less well characterized than those of ghrelin) using the plasma from 19 elderly research participants aged 60 or older, without obesity or dementia; they all had Mini-Mental State Examination (MMSE) scores higher than 25 (Supplemental Table 1; supplemental material available online with this article; https://doi.org/10.1172/jci.insight.166175DS1). Correlation analyses showed a negative relationship between age and plasma ghrelin (Figure 1A), whereas total ghrelin (that is, ghrelin and des-acyl ghrelin) displayed no association with age (Figure 1B). Contrary to ghrelin, plasma LEAP2 increased with age (Figure 1C). The changes in ghrelin and LEAP2 resulted in an age-dependent augmentation of the LEAP2/ghrelin molar ratio (Figure 1D). Given evidence in the literature supporting LEAP2 as a key determinant of ghrelin resistance (33), this newly observed age-dependent LEAP2/ghrelin imbalance is predicted to promote more robust ghrelin resistance over the course of normal aging in adults. Notably, a previous study utilizing plasma samples from adults with a wide range of BMIs determined negative correlations of ghrelin levels and positive correlations of LEAP2 levels and LEAP2/ghrelin molar ratios with BMI and percent body fat in adults (32); these correlations of ghrelin and LEAP2 with BMI have been documented in several other studies as well (36, 37). In our cohort, which included participants with a much narrower range of BMI than that in the study conducted by Mani et al. (32), no correlations between BMI and age (Figure 1E), LEAP2 (Figure 1F), ghrelin (Figure 1G), or the LEAP2/ghrelin molar ratio (Figure 1H) were observed. Furthermore, neither plasma LEAP2, ghrelin, nor total ghrelin showed a correlation with plasma GH (Figure 1, I–K), corroborating a previous report of the compromised effect of ghrelin signaling in aged participants, regardless of their ghrelin levels or metabolic status (23). Our findings suggest that the aging process is associated with both LEAP2 and ghrelin perturbations, which together cause a LEAP2/ghrelin imbalance relative to younger individuals.

### LEAP2/ghrelin imbalance is associated with cognitive impairment in elderly participants without dementia.

**Figure 2:**
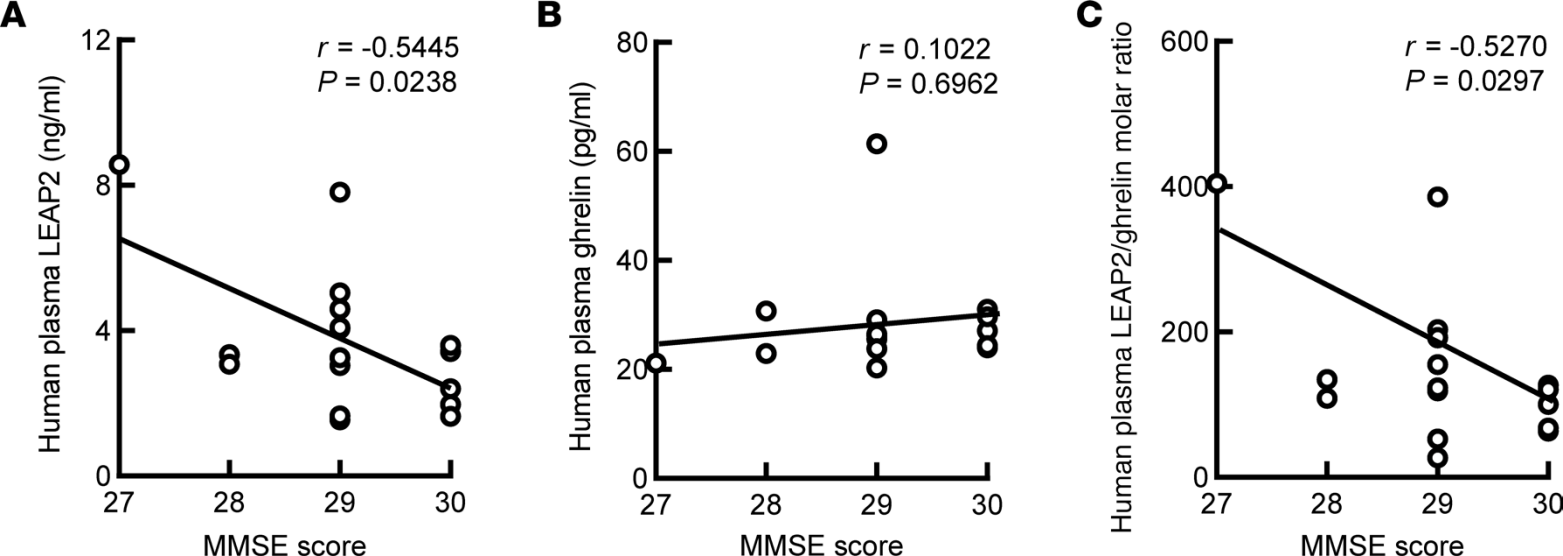
Negative association of LEAP2/ghrelin ratio with MMSE score in the elderly. (**A**–**C**) Correlation between MMSE score and plasma LEAP2 (**A**), plasma LEAP2/ghrelin ratio (**B**), and plasma ghrelin (**C**) were calculated using 2-tailed Pearson correlation coefficients. *n* = 17.

In view of the importance of ghrelin signaling to cognition (13, 16–18, 38), we examined the potential impact of the age-related LEAP2/ghrelin imbalance on cognition in our cohort of elderly participants without dementia. While circulating LEAP2 (Figure 2A) was negatively correlated with MMSE scores, which we considered a proxy of cognitive function as per Kvitting et al. (39), no correlation between ghrelin and MMSE score was detected (Figure 2B). Altogether, the LEAP2/ghrelin molar ratio (Figure 2C) was negatively correlated with MMSE scores. Of note, consistent with the reported lack of association between normal body weight and cognitive deficits in the elderly (40), we extended the analysis and found no correlation of BMI with MMSE scores (Supplemental Figure 1A), suggesting that a BMI within normal range does not have a profound impact on cognition. Moreover, no multicollinearity was present in a regression model to detect an association of MMSE scores with a combined LEAP2/ghrelin molar ratio and BMI (Supplemental Figure 1B) in our tested cohort, indicating a lack of interaction between LEAP2 dysregulation and BMI in promoting age-related cognitive decline. Therefore, given the critical role of ghrelin signaling in hippocampal physiology (20, 38, 41, 42), these findings imply a potential influence of age-related LEAP2/ghrelin imbalance as an independent risk on cognitive function.

### LEAP2 inhibits ghrelin-mediated hippocampal synaptic function.

**Figure 3:**
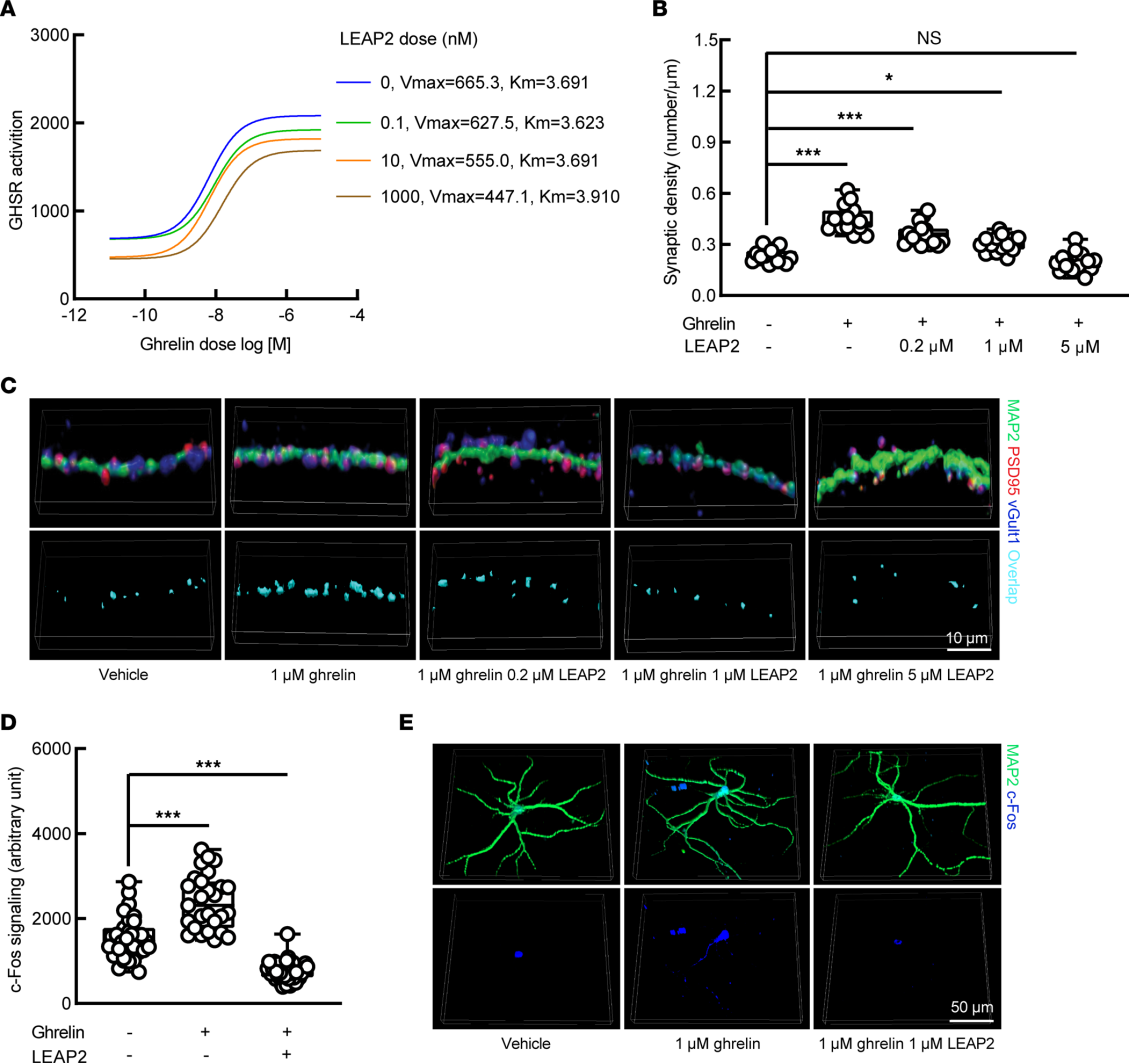
Inhibitory effect of LEAP2 on GHSR activity and hippocampal synaptic function. (**A**) The influence of LEAP2 on GHSR activation was examined by Tango β-arrestin recruitment assay. V_max_ and K_m_ were calculated using Michaelis-Menten equation. *n* = 8 repeats for each dose combination. (**B** and **C**) A suppressive effect of LEAP2 on synaptic density in cultured hippocampal neurons. *n* = 15 neurons in each group. (**C**) The representative images. Synapses were quantified as colocalized presynaptic marker (vGlut1, blue) and postsynaptic marker (PSD95, red). MAP2 (green) was used as a neuron marker. Scale bar: 10 μm. (**D** and **E**) Immunostaining of c-Fos in ghrelin- or LEAP2/ghrelin-treated cultured hippocampal neuron. *n* = 27–31 neurons in each group. (**E**) The representative images. Scale bar: 50 μm. One-way ANOVA followed by Bonferroni post hoc analysis. *P < 0.05, ***P < 0.001.

Although the inhibitory effects of LEAP2 on ghrelin-induced GHSR activation in heterologous expression systems (31, 38) and on ghrelin-mediated hypothalamic neuron activity in ex vivo (32) and in vivo (43) settings have been previously defined, and although important roles for GHSR constitutive activity to maintain the synaptic content of AMPA-type glutamate receptors (44) and impair inhibitory neurotransmission have been identified within cultured hippocampal neurons (12), it remains undetermined whether LEAP2 suppresses ghrelin-mediated hippocampal synaptic function. To address this question, we examined synaptogenesis in primary cultured hippocampal neurons. The efficacy of LEAP2 in suppressing ghrelin-mediated GHSR activation was confirmed by using a Tango β-arrestin recruitment assay system (45). With increasing doses, LEAP2 decreased the maximum rate of reaction when all active sites of receptor are bound to substrate (V_max_) but had no impact on the substrate concentration at which reaction rate is 50% of V_max_ (K_m_) for ghrelin-mediated GHSR activation (Figure 3A), indicating that LEAP2 is a noncompetitive inhibitor that allosterically modulates GHSR activation. This finding corroborates previous reports (29, 46) and endorses our further testing on the response of primary hippocampal neuron cultures to ghrelin in the presence or absence of LEAP2. Compared with vehicle treatment, hippocampal neurons treated with ghrelin showed increased synaptic density (Figure 3, B and C), which is in agreement with the reported effect of ghrelin on promoting synaptic plasticity (13, 17, 38). However, ghrelin-elicited hippocampal neuronal synaptogenesis was diminished by LEAP2 in a dose-dependent manner (Figure 3, B and C). Moreover, ghrelin-mediated c-Fos expression in hippocampal neurons was lessened by the addition of LEAP2 (Figure 3, D and E), further supporting an inhibitory impact of LEAP2 on ghrelin-mediated excitatory neuronal activity (47). Therefore, these in vitro observations suggest a dose-dependent inhibitory effect of LEAP2 elevation on ghrelin-mediated increases in synaptic density and neuronal activation within the hippocampus, and they further warrant the extension of our study to an in vivo setting, which holds more pathophysiological relevance to aging.

### LEAP2/ghrelin imbalance blunts hippocampal ghrelin signaling in aged mice.

**Figure 4:**
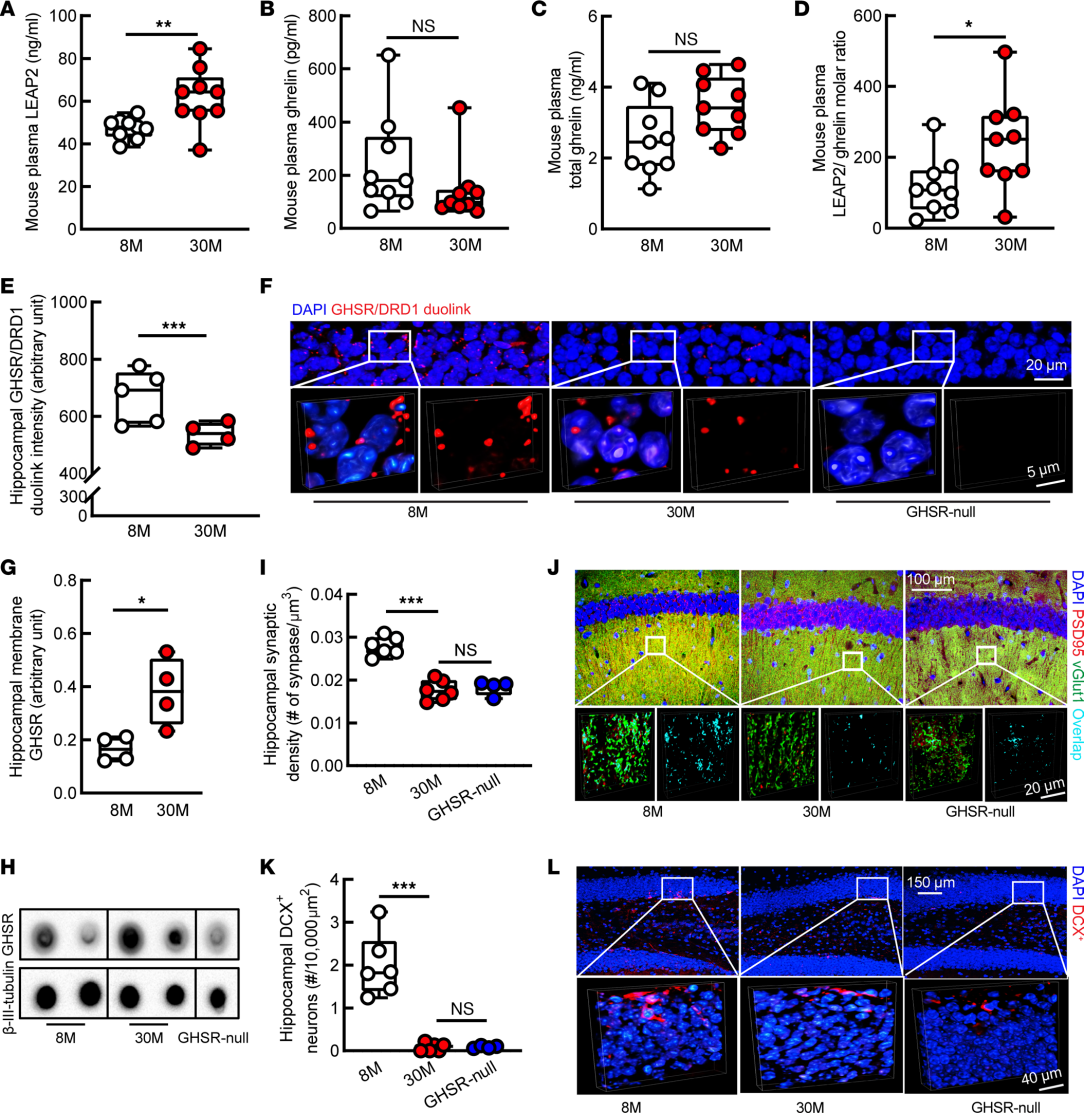
Hippocampal ghrelin signaling deregulation and pathology in old mice. (**A**) Thirty-month-old (30M) mice showed increased plasma LEAP2 compared with 8M mice. (**B** and **C**) Plasma ghrelin (**B**) or total ghrelin (**C**) showed no change. (**D**) Plasma LEAP2/ghrelin molar ratio increased in 30M mice. (**A**–**D**) *n* = 9 mice per group. (**E** and **F**) Hippocampal GHSR/DRD1 heterodimerization by Duolink PLA assay. 8M, *n* = 5; 30M, *n* = 4; GHSR-null, *n* = 2. (**F**) Representative images. Scale bar: 20 μm (inset, scale bar: 5 μm). GHSR-null mice were used to verify Duolink PLA assay. (**G** and **H**) Hippocampal membrane–bound GHSR expression by membrane blot. β-III Tubulin was used as the loading control. 8M, *n* = 4; 30M, *n* = 4; GHSR-null, *n* = 2. (**H**) The representative images. GHSR-null mice were used to verify the specificity of membrane blot. The dots were run on the same membrane but were noncontiguous. (**I** and **J**) Synaptic density of hippocampal CA1 region was analyzed by colocalization of presynaptic marker (vGlut1) and postsynaptic marker (PSD95). 8M, *n* = 6; 30M, *n* = 6; GHSR-null, *n* = 4. (**J**) The 3D-reconstructed representative images. Scale bar: 100 μm (inset, scale bar: 20 μm). (**K** and **L**) The number of doublecortin^+^ (DCX^+^) neurons were counted in the dentate gyrus. 8M, *n* = 6; 30M, *n* = 6; GHSR-null, *n* = 4. (**L**) The 3D-reconstructed representative images. Adult neurogenesis was determined by DCX^+^ staining. Scale bar: 150 μm (inset, scale bar: 40 μm). GHSR-null, 8-month-old GHSR-null mice. Unpaired Student’s *t* test was used in **A**–**E** and **G**; 1-way ANOVA followed by Bonferroni post hoc analysis was used in **I** and **K**. *P < 0.05, **P < 0.01, ***P < 0.001. Sagittal brain slices that were 0.8–1 mm lateral to the medial plane were used in **E** and **F**, and slices 1–1.2 mm were used in **I**–**L**.

Cognitive decline and hippocampal lesions during aging have been repeatedly identified in mice over 22 months old (old mice) (48, 49). To determine whether the age-related LEAP2/ghrelin imbalance observed in our cohort of aged human participants is recapitulated in aged mice, plasma LEAP2 and ghrelin levels were measured in male and female C57BL/6NJ mice at 8 and 30 months old. Despite comparable body weights (stratified by sex) between the 2 groups (Supplemental Figure 2A), 30-month-old mice exhibited increased plasma LEAP2 as compared with their middle-aged, 8-month-old counterparts (Figure 4A). There was not an observed age effect on plasma ghrelin (Figure 4B) or total ghrelin (Figure 4C). Altogether, these observed age-related changes in LEAP2 and ghrelin resulted in a LEAP2/ghrelin molar ratio that was, on average, approximately 34% higher in 30-month-old mice as compared with 8-month-old mice (Figure 4D). These changes were not dependent on sex (Supplemental Figure 2, B and C).

To further characterize the impact of the aging-related elevation in the LEAP2/ghrelin molar ratio on the hippocampus, we examined ghrelin signaling–related hippocampal functions in aged mice. Ghrelin activates GHSR, resulting in a subsequent physical interaction between GHSR and dopamine receptor D1 (DRD1) to modulate hippocampal synaptic activity (16). Therefore, we first examined hippocampal GHSR/DRD1 heteromers using a Duolink Proximity Ligation Assay (PLA) (17) in mice at 8 and 30 months old. Compared with their middle-aged counterparts, old mice exhibited decreased density of GHSR/DRD1 PLA^+^ dots in their hippocampi (Figure 4, E and F). By immunofluorescence staining, increased expression of GHSR (Supplemental Figure 3A) and DRD1 (Supplemental Figure 3B) was observed in the hippocampi of aged mice, indicating that the age-related reduction in hippocampal GHSR/DRD1 complexes is not likely to be a result of GHSR and/or DRD1 deficiency. Moreover, aged mice exhibited increased membrane-bound GHSR in their hippocampi (Figure 4, G and H). Because GHSR internalizes after activation (50), increased membrane-bound GHSR implies suppressed hippocampal GHSR activation in old mice. In accordance with disrupted GHSR/DRD1 heteromerization, aged mice showed reduced synaptic density in their hippocampal CA1 region (Figure 4, I and J), an aging-sensitive brain area, the dysfunction of which is associated with age-related cognitive deficits (51). Our observations of LEAP2-inhibited GHSR/DRD1 interaction and the reported disrupted GHSR and DRD2 heteromerization by LEAP2 or LEAP2 fragments (31) support a suppressive effect of LEAP2 on GHSR’s interactions with dopamine receptors. Next, in view of a critical role of ghrelin signaling in promoting hippocampal neurogenesis (18, 20, 21), we assessed neurogenesis within hippocampal slices from mice at 8 and 30 months old by staining for Doublecortin (DCX) (17). Compared with 8-month-old mice, the 30-month-old mice exhibited decreased DCX^+^ neurons in their dentate gyrus (DG) (Figure 4, K and L), suggesting impaired hippocampal neurogenesis with aging. These aging-related hippocampal changes in 30-month-old C57BL/6NJ mice — including reduced hippocampal CA1 synaptic density (Figure 4, I and J) and reduced hippocampal neurogenesis (Figure 4, K and L), alongside loss of hippocampal GHSR/DRD1 interactions (Figure 4, E and F) — resembled hippocampal changes observed in 8-month-old GHSR*-*null littermates. These similarities between 30-month-old C57BL/6NJ mice and 8-month-old GHSR-null mice, coupled with the observed age-related increase in the plasma LEAP2/ghrelin molar ratio in C57BL/6NJ mice, are further evidence suggesting involvement of GHSR signaling dysregulation — and, in particular, ghrelin resistance resulting from LEAP2/ghrelin imbalance — in promoting age-related hippocampal pathology.

### Downregulation of LEAP2 reverses cognitive deficits in aging mice.

**Figure 5:**
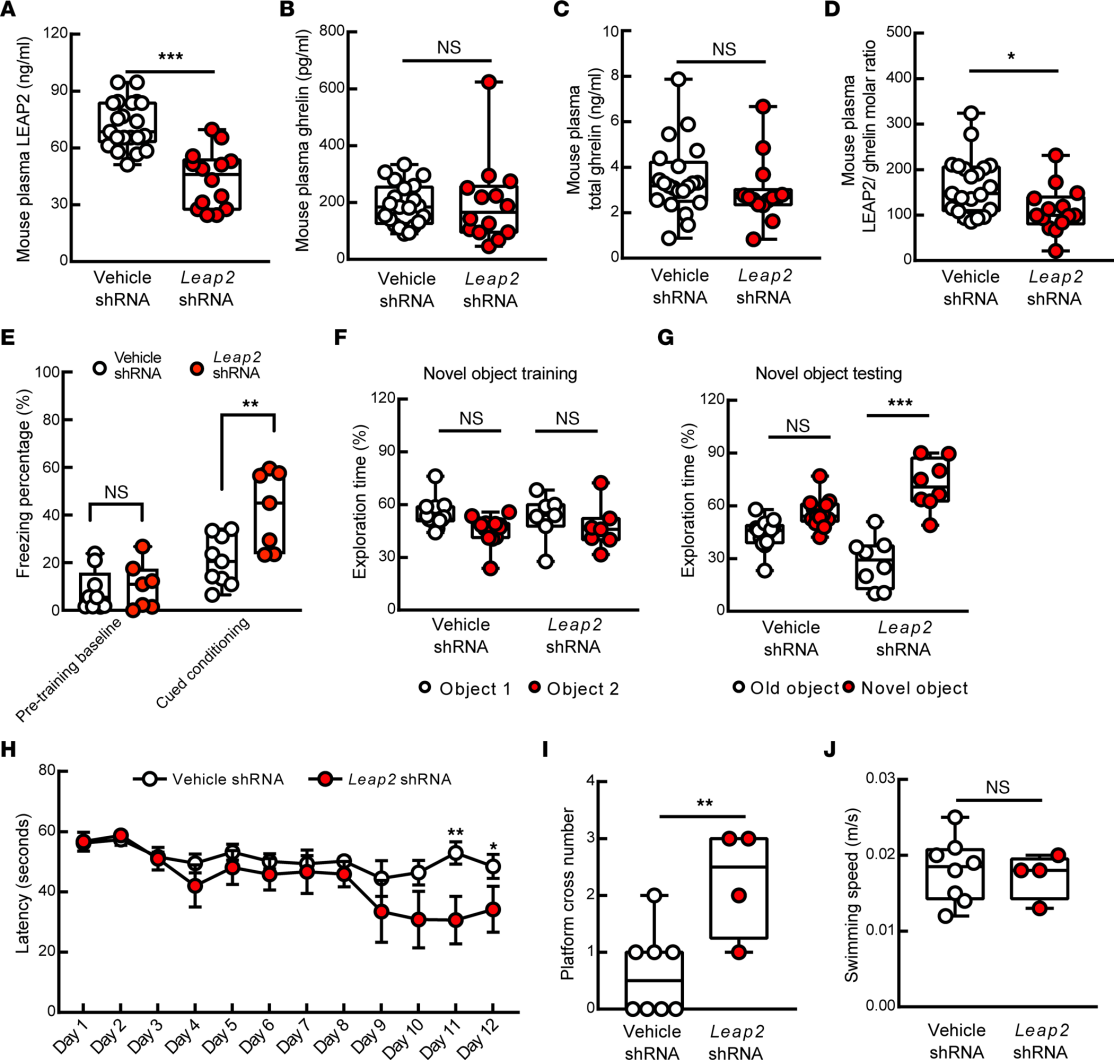
Protective effect of LEAP2 downregulation on cognitive performance of old mice. (**A**) Plasma LEAP2 decreased in *Leap2* shRNA–injected mice. (**B** and **C**) Plasma ghrelin or total ghrelin levels showed no change in *Leap2* shRNA–treated mice. (**D**) Plasma LEAP2/ghrelin ratio in old mice with or without LEAP2 modulation. (**A**–**D**) Vehicle shRNA, *n* = 21; *Leap2* shRNA, *n* = 14. (**E**) Mouse freezing percentage in old mice with or without LEAP2 modulation. Vehicle shRNA, *n* = 9; *Leap2* shRNA, *n* = 7. (**F** and **G**) Mouse novel object recognition in old mice with or without LEAP2 modulation. (**F**) Training phase with 2 identical objects. (**G**) Testing phase with 1 familiar object and 1 novel object. Vehicle shRNA, *n* = 13; *Leap2* shRNA, *n* = 8. (**H**–**J**) Spatial navigation of old mice with or without LEAP2 modulation. (**H**) Spatial learning ability represented by the latency to find the hidden platform during the learning phase. (**I**) Spatial reference memory represented by platform cross number in the probing phase. (**J**) Swimming speed during probing phase. Vehicle shRNA, *n* = 8; *Leap2* shRNA, *n* = 4. Unpaired Student’s *t* test. *P < 0.05, **P < 0.01, ***P < 0.001.

To establish a direct link between LEAP2 dysregulation and age-related cognitive decline, we employed lentiviral shRNA silencing of *Leap2* to downregulate LEAP2 production in aged mice. Due to the importance of the LEAP2/ghrelin balance in physiology (33), we optimized the load of lentiviral vectors to avoid excess loss of LEAP2. *Leap2* shRNA–challenged aged mice demonstrated reduced plasma LEAP2 as compared with their age- and sex-matched lentiviral control vector–treated (vehicle-treated) counterparts (Figure 5A). The mRNA (Supplemental Figure 4A) and protein expression (Supplemental Figure 4B) of LEAP2 were consistently decreased in the liver after *Leap2* shRNA treatment. As a result of unaffected plasma ghrelin (Figure 5B) and total ghrelin (Figure 5C), *Leap2* shRNA–treated 30-month-old mice exhibited a LEAP2/ghrelin molar ratio comparable with that in middle-aged, 8-month-old mice (Figure 5D and Figure 4D; 8-month-old mice [ratio of 118.7 ± 27.1] versus *Leap2* shRNA–treated 30-month-old mice [ratio of 111.1 ± 13.58]; *P* = 0.7838). Corroborating a previous study on standard chow-fed LEAP2-KO mice (33), LEAP2-manipulated mice did not exhibit change in their food (Supplemental Figure 5A) or water (Supplemental Figure 5B) consumption. Moreover, LEAP2 downregulation did not impact blood glucose (Supplemental Figure 5C) or free fatty acids (Supplemental Figure 5D) in the fasted state. To examine the impact of LEAP2/ghrelin rebalance on cognitive performance, we performed behavioral tests including a cued-fear conditioning test, a novel object recognition (NOR) test, and the Morris water maze to assess hippocampus-associated contextual memory (52), recognition memory (53), and spatial reference memory (54), respectively. In the cued-fear conditioning test, despite a comparable pretraining baseline freezing percentage (Figure 5E), the posttraining freezing percentage was increased in LEAP2-downregulated aged mice as compared with their vehicle-exposed counterparts (Figure 5E). Furthermore, although both vehicle- and *Leap2* shRNA–treated mice showed no difference in exploring 2 identical objects during the training phase (Figure 5F), *Leap2* shRNA–treated mice displayed improved object recognition memory demonstrated by spending longer time on the novel object during the testing phase (Figure 5G). Lastly, LEAP2-manipulated mice exhibited enhanced spatial learning (Figure 5H) and memory (Figure 5I) in the Morris water maze test with unaffected mouse swimming speed (Figure 5J) in comparison with their vehicle-treated counterparts. These results support a contribution of LEAP2/ghrelin imbalance to aging-related cognitive deficits.

### Downregulation of LEAP2 alleviates hippocampal changes in aging mice.

**Figure 6:**
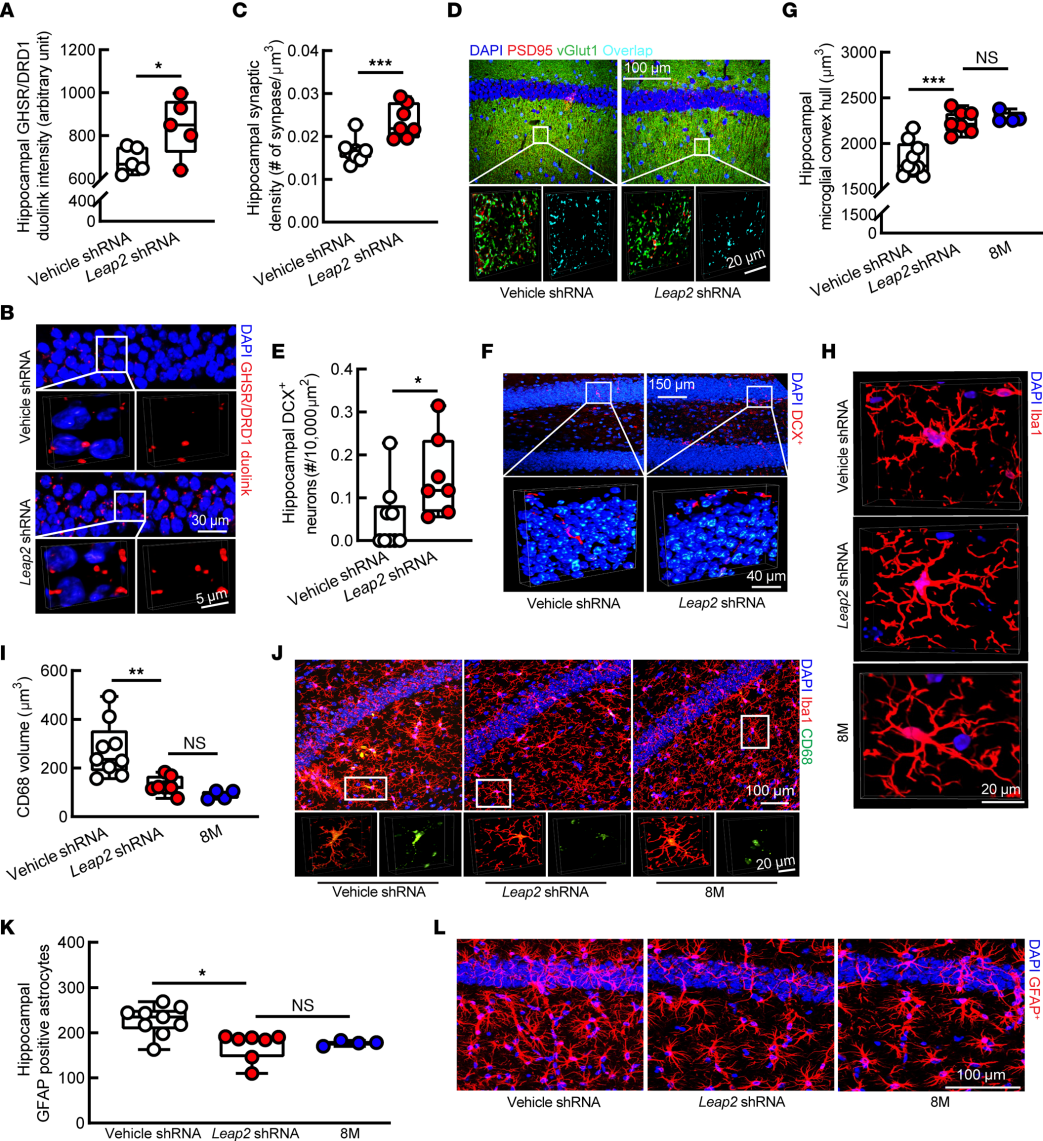
Mitigated hippocampal changes in LEAP2-downregulated old mice. (**A** and **B**) Hippocampal GHSR/DRD1 PLA^+^ dots intensity in vehicle or Leap2 shRNA-treated mice. Vehicle or LEAP2 shRNA, *n* = 5. (**B**) Representative images. Scale bar: 30 μm (inset, scale bar: 5 μm). (**C** and **D**) Hippocampal CA1 region synaptic density of vehicle or Leap2 shRNA–treated mice. Vehicle shRNA, *n* = 9; Leap2 shRNA, *n* = 7. (**D**) Representative images. Scale bar: 100 μm (inset, scale bar: 20 μm). (**E** and **F**) DCX^+^ neurons in the dentate gyrus of vehicle or Leap2 shRNA–treated mice. Vehicle shRNA, *n* = 9; Leap2-shRNA, *n* = 7. (**F**) Representative images. Scale bar: 150 μm (inset, scale bar: 4 μm). (**G** and **H**) Hippocampal CA1 region microglial convex hull of vehicle or Leap2 shRNA–treated mice and untreated 8M mice. Vehicle shRNA, *n* = 9; Leap2 shRNA, *n* = 7; 8M *n* = 4. (**H**) Representative images. Scale bar: 20 μm. (**I** and **J**) Microglial CD68 volume in the hippocampal CA1 region of vehicle or Leap2 shRNA–treated mice and untreated 8M mice. Vehicle shRNA, *n* = 9; Leap2 shRNA, *n* = 7; 8M, *n* = 4. (**J**) Representative images. Scale bar: 100 μm (inset, scale bar: 20 μm). (**K** and **L**) GFAP^+^ astrocyte density in the hippocampal CA1 region of vehicle or Leap2 shRNA–treated mice and untreated 8M mice. Vehicle shRNA, *n* = 9; Leap2 shRNA, *n* = 7; 8M *n* = 4. (**A**) Representative images. Activated astrocytes labeled with GFAP antibody. Scale bar: 100 μm. 8M, 8-month-old mice. Unpaired Student’s *t* test in **A**–**F**; 1-way ANOVA followed by Bonferroni post hoc analysis in **G**–**L**. *P < 0.05, **P < 0.01, ***P < 0.001. Sagittal brain slices that were 0.8–1 mm lateral to the medial plane were used in **A** and **B** and 1–1.2 mm in **E**–**L**.

To determine whether the improved cognitive function in LEAP2-downregulated aged mice is associated with restored hippocampal ghrelin signaling, we first examined hippocampal GHSR/DRD1 interaction. PLA Duolink assays showed increased GHSR and DRD1 complexes in LEAP2-downregulated aged mice (Figure 6, A and B) without affecting the expression of hippocampal GHSR (Supplemental Figure 6A) or DRD1 (Supplemental Figure 6B), as determined by immunostaining. Furthermore, we examined synaptic density in the hippocampal CA1 region by immunostaining and observed mitigated synaptic loss in *Leap2* shRNA–treated aged mice (Figure 6, C and D). In addition, downregulation of LEAP2 displayed a restoration of hippocampal neurogenesis in aged mice (Figure 6, E and F). Moreover, neuroinflammatory damages contribute to hippocampal aging (55), and previous studies have implicated an anti-neuroinflammatory effect of ghrelin signaling in the brain (56, 57). By immunostaining, the vehicle-treated old mice showed decreased microglial convex hull (Figure 6, G and H), increased microglial CD68 volume (Figure 6, I and J), and augmented glial fibrillary acidic protein^+^ (GFAP^+^) astrocytes (Figure 6, K and L) in their hippocampi as compared with middle-aged mice at 8 months old, indicating that enhanced hippocampal neuroinflammation is an age-related effect. In contrast, age-related neuroinflammation was attenuated by LEAP2/ghrelin rebalancing (Figure 6, G–L). Together with improved cognition, these observations indicate that rebalancing the LEAP2/ghrelin molar ratio to one that is reminiscent of younger individuals upregulates hippocampal GHSR signaling and rescues hippocampal functions in aging.

## Discussion

Although individuals of the same chronological age may display variations in cognitive performance, advanced age is generally associated with cognitive deficits such as impaired memory, slowed processing speed, and compromised reasoning capacity as well as defected executive functions (58). Such age-related cognitive deficits differ from dementia symptoms both quantitatively and qualitatively but affect the daily life quality of many older adults (59). The hippocampus is a pivotal structure for cognition and social behavior (60). Clinical and basic research has linked hippocampal dysfunction, including synaptic and neuronal degeneration, to the development of age-associated memory loss (61–64). In this study, we have identified an unprecedented role of disrupted LEAP2/ghrelin balance in the development of hippocampal synaptic deficits and cognitive decline during aging. The elevated LEAP2/ghrelin molar ratio accompanying aging blunted hippocampal ghrelin signaling, resulting in suppressed hippocampal synaptic function, impaired hippocampal neurogenesis, and neuroinflammatory damage, culminating in cognitive deficits. Just as an elevated LEAP2/ghrelin molar ratio has been proposed as a contributing factor to the ghrelin resistance associated with obesity (32), we now propose a “hippocampal ghrelin resistance” hypothesis of normal cognitive aging. Given the direct deleterious impact of LEAP2 dysregulation on ghrelin’s function in promoting hippocampal fitness, modulation of the LEAP2/ghrelin balance, therefore, holds promise to rejuvenize cognitive functions or, at very the least, to delay cognitive decline in aging.

Indeed, aging is a complex process that involves changes in multiple systems that may cumulatively cause damage to the functions of the brain. A critical scientific issue that should not be overlooked is the close relationship between metabolic status and the regulation of LEAP2 and ghrelin (32). It is worth mentioning that ghrelin signaling promotes food intake and suppresses fat mobilization (65–67). Suarez and colleagues have recently determined the importance of the hippocampus-hypothalamus axis to ghrelin-mediated increases in meal size (68), supporting a sophisticated relationship between hippocampal ghrelin signaling, memory, and feeding behavior (69). However, the impact of LEAP2 on meal ingestion and metabolism is complicated by mixed results from previous studies. In contrast to a previous report that administration of LEAP2 peptide enhances food intake in human males (70), the inhibitory effect of LEAP2 against ghrelin-induced food intake was observed in high-fat diet but not standard chow–fed rats with LEAP2 overexpression in the arcuate nucleus (ARC) of the hypothalamus (71). Moreover, a recent study found that genetic depletion of *Leap2* only increases food intake in female mice fed a high-fat diet, while mice with LEAP2 deficiency did not display any genotypic effect on their eating behavior, body weight, or fasting blood glucose when exposed to a standard chow diet (33). These findings are in agreement with our observation of no change in food and water ingestion in LEAP2-downregulated mice. Although the precise mechanisms of LEAP2-mediated feeding regulation so far remain unclear, it is likely that LEAP2 exerts its antiorexigenic effects most prominently in an overnutrition state, and this assessment is supported by a disrupted LEAP2/ghrelin balance toward an increased LEAP2/ghrelin molar ratio in obese participants with a BMI higher than 30 kg/m^2^ and a postprandial LEAP2 elevation in obese women but not in those within a normal BMI range (32). In the current study, we did not observe an association of LEAP2, ghrelin, or the LEAP2/ghrelin molar ratio with the BMI of the tested cohort. One potential explanation of the limited impact of body weight is that none of the participants in current study carried a diagnosis of obesity and, instead, had BMIs within a lower and relatively narrow range. In addition, participants with metabolic disorders such as diabetes, hyperlipidemia, and hypercholesterolemia, as well as thyroid and hypothalamic disorders that may potentially affect LEAP2 regulation (72, 73), were excluded. Whether this age-related upward shift in the LEAP2/ghrelin balance occurs as a natural defense against potentially deleterious, age-associated metabolic changes — perhaps at the expense of cognitive decline — is an interesting thought experiment. Certainly, energy dysmetabolism is a well-documented change with advanced age that contributes to life-threatening conditions such as cardiovascular diseases (74) and stroke (75). In this context, our findings raise a pivotal question of whether potential future therapies that lower plasma LEAP2/ghrelin molar ratios of seniors to levels present in younger adults as a strategy to improve cognition would result in unintended metabolic changes, such as increased adiposity, especially in the obese participants. Furthermore, the engagement of the hippocampus in ghrelin-related food intake (68) also inspires a further in-depth investigation of a feedback loop involving cognition and metabolism.

Another critical issue that merits discussion is whether LEAP2 dysregulation is mechanistically associated with increased risk of Alzheimer’s dementia. Although previous studies have demonstrated a contribution of GHSR deactivation to hippocampal pathology in AD (17, 76), it should be noted that amyloid β (Aβ), a crucial pathological factor in AD, underlies hippocampal GHSR dysfunction in an AD context (17). In contrast, the amount of brain Aβ in normal aging brains is not as drastic as in the setting of AD amyloidosis. Although we cannot completely refute a possible contribution of Aβ to hippocampal ghrelin signaling dysfunction in aging brains, this issue is complicated by the close association of an increased LEAP2/ghrelin molar ratio with chronological age, as the incidence of AD is augmented with age (77). In our study, the participants, even those older than 85 years, did not exhibit symptoms that merited a diagnosis of dementia. In view of the contribution of glucose and lipid dysmetabolism to the development of AD (78), the increased LEAP2/ghrelin molar ratio in older adults might be a systemic change to accommodate metabolic changes during aging and, thus, prevent AD and other age-related conditions. This is supported by a study showing a protective effect of ghrelin depletion against aging-related obesity and muscle loss (79). However, corroborating previous findings of a beneficial effect of ghrelin signaling against the aging process (26, 80), an alternative interpretation of the results is that the exacerbation of abnormal LEAP2 levels, if left untreated, may worsen hippocampal aging and the age-related cognitive decline, eventually leading to severe consequences such as AD. These questions may be answered by further examination of the LEAP2/ghrelin molar ratio in patients with AD. Although we did not observe any change in glucose and lipids in LEAP2-modulated aged mice, these concerns remind us to pay attention to the management of metabolism when applying long-term LEAP2 modulation to future clinical trials for the treatment of age-related cognitive decline.

Lastly, our observations of an association between LEAP2 dysregulation and advanced aging have raised an interesting question of whether LEAP2 and ghrelin imbalance constitutes a discernible change accompanying aging. A previous study reported a lack of correlation between age and ghrelin or LEAP2 in children aged 3 to 12 years (35), and this contrasts with our observations of age-related changes of LEAP2 and ghrelin in older adults. Such a difference in ghrelin and LEAP2 between children and older adults may indicate an age-dependent distinct regulation of these metabolism-related hormones. Of note, increased LEAP2 and decreased ghrelin have been associated with puberty stages in female children and adolescents aged 3 to 17 years (81). The unaffected food intake in the studied participants seems to suggest an intertwined relationship between ghrelin, LEAP2, and other metabolic hormones as well as reproductive hormones (81). Intriguingly, previous studies on fully developed adults reported a LEAP2/ghrelin molar ratio of 48:1 in healthy young males between 18 and 25 years of age (70) and a range of plasma LEAP2/ghrelin molar ratios between 45:1 and 100:1 in nonobese healthy middle-aged adults at a median age of 35 (32). In our tested cohort of older adults, the average LEAP2/ghrelin molar ratio reached 153.4 ± 23.4 to 1, supporting that an increased LEAP2/ghrelin molar ratio is an age effect. Furthermore, LEAP2 and ghrelin alterations with advanced aging may also constitute an example of differences in metabolic regulation in different stages of the lifecycle. Notably, we found that aged participants with preserved cognition demonstrated by a ceiling MMSE score of 30 had a LEAP2/ghrelin molar ratio of 95.8 ± 13.1 to 1, which falls into the range of LEAP2/ghrelin ratios in middle-aged adults. This observation endorses an interaction between LEAP2 and cognition during normal aging and further echoes our findings in mice of the protective effects of the restoration of the LEAP2/ghrelin molar ratio to levels present in middle-aged mice. Therefore, the age-related changes of LEAP2 may precipitate cognitive decline and function as an indicator of memory disturbances during aging.

In summary, our findings support a LEAP2-induced “ghrelin resistance” hypothesis of hippocampal dysfunction and memory loss during advanced aging and endorse a therapeutic potential of modulating LEAP2 levels as a means of treating age-related cognitive decline. It could be argued whether supplementation of ghrelin is another possible avenue to restore the LEAP2/ghrelin balance for the treatment of age-associated cognitive deficits in view of the neurotrophic functions of ghrelin; however, previous studies showed that a high dose of ghrelin has an unexpected adverse impact on the hippocampus-related cognition (82). Accordingly, the restoration of the LEAP2/ghrelin molar ratio such that both LEAP2 and ghrelin levels are restored to levels present in younger adults may have its advantages. Further in-depth mechanistic studies are needed to determine the pathways driving LEAP2 abnormalities during normative aging and its potential pathophysiological role during aging. Another limitation of the current study is the small cohort size due to our stringent inclusion and exclusion criteria. In our future study, we will recruit a larger cohort with a wider range of BMI and itemized MMSE scores to evaluate the diagnostic capacity of LEAP2/ghrelin imbalance in identifying hippocampus-related cognitive deficits during normal aging. Lastly, given the age-associated damages in the blood-brain barrier (BBB) (83, 84), it is likely that advanced aging renders the hippocampus vulnerable to LEAP2 dysregulation. In this context, the intertwined relationship between hippocampal function and metabolic regulation (69), thus, warrants an investigation into the interaction of BBB integrity, LEAP2 modulation, and cognitive performance to deepen our understanding of aging biology and to advance the development of LEAP2-targeting therapies for the treatment of memory loss accompanying aging. Nevertheless, this proof-of-concept study indicates that LEAP2 dysregulation is part of the aging process and that it may play a role in the fall of hippocampal synaptic activity in the senior population. The simplest interpretation of our data is that LEAP2 abnormality is an age effect and that redressing LEAP2/ghrelin balance may benefit hippocampal synaptic fitness and improve memory in the elderly.

## Methods

### Patients.

Human plasma samples were requested from the University of Kansas Alzheimer’s Disease (KUAD) center under a KU Medical Center-approved protocol supported by an NIH grant (P30 AG035982). To generate these plasma samples, blood was collected by phlebotomy, placed into ice-cold EDTA-treated tubes with a protease inhibitor cocktail (MilliporeSigma, 20-201), and centrifuged at 1,500*g* at 4°C for 15 minutes. The plasma samples were stored at –80°C until assay. Of note, although the use of EDTA, protease inhibitors, and cold temperature all represent key steps in stabilizing the octanoylated form of ghrelin that binds to GHSR, these plasma samples were not acidified by the addition of HCl (unlike the mouse plasma samples used below for ghrelin assays), and this could result in some ghrelin degradation (85, 86).

Participants older than 60 and with MMSE scores ≥ 27 were recognized as nondemented normal aging (87). Participants with metabolic and endocrine disorders including hypothalamic disorders, diabetes, familial hypercholesterolemia, hyperlipidemia, hyperthyroidism, hypothyroidism, inflammatory diseases, and liver and renal disorders, as well as those under treatment of weight-control drugs, lipid-lowering agents, and amylin mimetics, were excluded from the study. Human demographic information was collected by the KUAD center.

### Animal studies.

C57BL/6NJ mice were originally obtained from The Jackson Laboratory. GHSR*-*null mice, which lack GHSR as a result of an inserted transcriptional blocking cassette within the *Ghsr* gene (88), were from a colony maintained at the University of Texas Southwestern Medical Center. GHSR*-*null mice on a C57BL/6N genetic background were backcrossed with C57BL/6NJ mice at least 10–12 times to generate GHSR*-*null mice on a C57BL/6NJ genetic background, which were used in this study. Genotypes of animals were confirmed using PCR.

Mouse whole blood samples from submandibular blood collection after 8 hours of fasting were put into ice-cold EDTA-treated tubes with a protease inhibitor cocktail (MilliporeSigma, 20-201). Plasma was prepared by centrifuging the whole blood sample for 15 minutes at 1,500*g* at 4°C for 15 minutes. Aliquots of plasma destined for ghrelin assay were further processed by adding HCl to a final concentration of 0.1N HCl and stored in –80°C for later use.

### Human plasma ELISA tests.

The plasma samples were subjected to the following tests using commercially available kits: total ghrelin ELISA (MilliporeSigma, EZGRT-89K), acylated ghrelin ELISA (MilliporeSigma, EZGRA-88K), and liver-enriched antimicrobial peptide 2 (LEAP2) ELISA (Phoenix Pharmaceuticals, EK-075-50). All tests were performed following standard protocols.

### Mice ELISA and chemical tests.

Eight- to 10-month-old C57BL/6NJ mice, 30-month-old C57BL/6NJ mice, and *Leap2* shRNA–injected 30-month-old C57BL/6NJ mice were subjected to the following tests using plasma acidified with 0.1N HCl: total ghrelin ELISA (MilliporeSigma, EZRGRT-91K) and acylated ghrelin ELISA (MilliporeSigma, EZRGRA-90K) as well as the following tests using plasma without HCl: LEAP2 ELISA (Phoenix Pharmaceuticals, EK-075-50), glucose quantitation (Thermo Fisher Scientific, TR15421), and free fatty acid assay (Sigma-Aldrich, MAK044). Vehicle shRNA– and *Leap2* shRNA–injected 30-month-old C57BL/6NJ mouse livers were homogenized in RIPA buffer — 150 Mm NaCl (Thermo Fisher Scientific), 0.1% SDS (Thermo Fisher Scientific), 0.1% NP-40 (Fluka), 0.1% sodium deoxycholate (MilliporeSigma), protease inhibitor cocktail (MilliporeSigma, 20-201), and 50 mM Tris (Thermo Fisher Scientific; pH 8.0). Hepatic homogenates were centrifuged for 10 minutes at 4°C at 12,000*g*, and supernatants were then proceeded to LEAP2 ELISA (Phoenix Pharmaceuticals, EK-075-50). All tests were performed following standard protocols.

### Real-time PCR.

Vehicle shRNA– and *Leap2* shRNA–injected 30-month-old C57BL/6NJ mouse hepatic mRNA was extracted using ZYMO Quick-RNA Microprep Kits (catalog R1050). Hepatic mRNA was converted to cDNA using TAKARA PrimeScript RT Master Mix (catalog RR036B). *Leap2* mRNA expression was examined using the following primer pair: *mLeap2* forward: 5′-GCTGCTGGGTCAGGTCAATAG-3′, *mLEAP2* reverse: 5′-CCGGGATCTCTTTGCTGAAC-3′. Real-time PCR was performed and analyzed with an ABI Stepone Plus RT-PCR machine.

### Tango β-arrestin recruitment assay.

GHSR activity was examined using the Tango β-arrestin recruitment assay that was developed by B.L. Roth (89). HTLA cells stably expressing a tTA-dependent luciferase reporter and a β-arrestin2-TEV fusion gene were a gift from B.L. Roth (University of North Carolina, Chapel Hill, North Carolina, USA). HTLA cells were maintained in DMEM with 10% FBS (Thermo Fisher Scientific), 100 U/mL penicillin/100 μg/mL streptomycin (Thermo Fisher Scientific), 2 μg/mL puromycin (Tocris Bioscience), and 100 μg/mL hygromycin B (Corning) at 37°C with 5% CO_2_. Cells were transfected with a GHSR-Tango plasmid expressing human GHSR and a vasopressin receptor (V_2_) tail (66293, Addgene) using the calcium-phosphate method and maintained for 24 hours prior to the next step. Transfected cells were transferred into a poly-D-lysine–coated 96-well plate (Corning) and maintained for 24 hours before treatment. Cells were then treated with an appropriate amount of human ghrelin peptide (Phoenix Pharmaceuticals, 031-30), LEAP2 peptide (Phoenix Pharmaceuticals, 075-40), or combinations of the 2 (LEAP2 was added 2 hours prior to ghrelin) at different ratios and doses for 24 hours to allow the expression of luciferase. Treated cells were incubated with Bright-Glo Luciferase Assay solution (Promega, E2610), and the signal was detected using a Biotek Neo2 microplate reader.

### Cell membrane isolation and membrane blotting.

Mouse hippocampal cell membranes were extracted using a previously published protocol (90). Mouse hippocampi were homogenized and incubated in ice-cold isolation buffer (50 mM Tris-HCl [pH 7.4], 1 mM MgCl_2_, 0.5 U/μL benzonase) for 10 minutes. Hippocampal cell membranes were isolated and washed for 3 times with 20 minutes of centrifugation at 16,500*g* at 4°C.

Purified hippocampal cell membranes were then fixed in 4% paraformaldehyde (PFA, MilliporeSigma) for 0.5 hours followed by 1-hour blocking — 5% donkey serum (Sigma-Aldrich), 0.3% Triton X-100 (Thermo Fisher Scientific), and PBS (pH 7.4). Membranes were incubated overnight in primary goat anti-GHSR1a antibody (Santa Cruz Biotechnology, sc-10359, 1:100) at 4°C. After washing with PBS-T (PBS containing 0.05% Tween-20) 3 times followed by centrifugation at 16,500*g* at 4°C for 20 minutes, membranes were incubated with anti–goat HRP–conjugated secondary antibody at room temperature for 1 hour. Cell membrane proteins were then extracted using urea buffer (50 mM Tris-HCl, 8M urea, 2% SDS, 10% glycerol [pH 6.8]). Cell membrane extracts were loaded onto a nitrocellulose membrane (Bio-Rad) and allowed to dry completely before imaging. The dried nitrocellulose membrane was subjected to imaging immediately using Bio-Rad Chemidoc Imaging System with signal developed using enhanced chemiluminescent substrate (ECL, Thermo Fisher Scientific). The membrane was reprobed with mouse anti–β-III tubulin (Proteintech, 66240, 1:1,000) to normalize protein levels.

### Immunocytochemistry.

Mouse brains were freshly dissected and fixed in 4% PFA overnight at 4°C. The frozen tissue sections were prepared as previously described (91). Primary cultured neurons on a Lab-Tek chamber slides were fixed in 4% PFA for 30 minutes at 37°C. After blocking (5% goat or donkey serum [Sigma-Aldrich], 0.3% Triton X-100 in PBS [pH 7.4]), brain slices or cultured neurons were incubated with primary antibodies against GHSR1a (Santa Cruz Biotechnology, sc-10359, 1:100), DRD1 (Abcam, ab81296, 1:200), PSD95 (Cell Signaling Technology [CST], 3450, 1:400), VGLUT1 (Synaptic Systems, 135304, 1:400), MAP2 (Sigma-Aldrich, M4403, 1:300), DCX (Santa Cruz Biotechnology, sc271390, 1:100), and c-Fos (Synaptic Systems, 226308, 1:400) in mixture or separately. After washing with PBS, the slices or neurons were probed with appropriate cross-adsorbed secondary antibodies conjugated to Alexa Fluor 488, Alexa Fluor 594, or Alexa Fluor 647 (Thermo Fisher Scientific, 1:500). Images were collected on a Nikon Ti2 confocal microscope. Mean intensity or volume of different staining were analyzed using Nikon-Elements Advanced Research software accordingly.

### Neuron culture and treatment.

Hippocampal neuron cultures were prepared as previously described (92). In brief, whole mouse hippocampi were dissected from P0 pups in cold HBSS. Cells were dissociated using 0.025% trypsin at 37°C for 15 minutes, followed by 10 times homogenization in ice-cold DMEM. Dissociated cells were then passed through a 100 μm cell strainer (Corning) and centrifuged for 5 minutes at 210*g*. The pellet was gently resuspended in neuron culture medium (Neurobasal A with 2% B27 supplement, 0.5 mM L-glutamine; Invitrogen) and plated on poly-D-lysine–coated (Sigma-Aldrich) Lab-Tek chamber slides (Nunc, no. 177445) with appropriate densities.

At 14 days in vitro (DIV), hippocampal neurons were exposed to synthetic mouse ghrelin (Phoenix Pharmaceuticals), LEAP2 (Phoenix Pharmaceuticals), or a mixture of ghrelin and LEAP2 for 5 minutes. The exposure was followed by immunostaining to examine the effects of Ghsr/Drd1 coactivation on synaptic function as described in the immunocytochemistry section.

### Duolink in situ assay.

Protein interactions between GHSR/DRD1 in mouse brain slices were detected using Duolink PLA detection kits (Sigma-Aldrich, DUO92008) following manufacturer’s instructions. The following primary antibodies were used: GHS-R1a (Santa Cruz Biotechnology, sc-10359, 1:100) and anti-DRD1 (Abcam, ab81296, 1:200). The specificity of antibodies to GHSR and DRD1 was validated as previously described (17). The following Duolink in Situ PLA Probes were used: anti–rabbit PLUS (Sigma-Aldrich, DUO92002) and anti–goat MINUS (Sigma-Aldrich, DUO92006). Images were collected on a Nikon Ti2 confocal microscope. The mean intensity of PLA^+^ signals was analyzed using Nikon-Elements Advanced Research software.

### Lentivirus packaging and mouse tail vein Leap2 shRNA injection.

Lentiviruses were packaged using packing vector psPAX2 and envelope vector pMD2.G (Addgene) in HEK293T cells that were maintained in DMEM with 10% FBS (Thermo Fisher Scientific) and 100 U/mL penicillin/100 μg/mL streptomycin (MilliporeSigma) at 37°C with 5% CO_2_. Lenti–mouse *Leap2* shRNA was purchased from Origene (no. TR519691). Lentivirus-expressing nontarget shRNA control (TRC2, SHC002, Sigma-Aldrich) was used as vehicle control. The titration of the lentiviruses was determined using the Lenti-X qRT-PCR Titration Kit (TAKARA, no. 631235).

Mouse hydrodynamic tail vein (HTV) injection was performed to increase delivery of lentiviruses to yield a higher level of hepatic LEAP2 knockdown as previous described (93). Briefly, Lenti–mouse *Leap2* shRNA or Lenti-vehicle shRNA viruses were diluted in sterilized Ringer’s solution (1.5 mM CaCl_2_, 5 mM KCl, 0.8 mM Na_2_HPO_4_ [pH 7.4]). In total, 7 ***×*** 10^7^ IFU of virus in 1.5 mL Ringer’s solution was delivered to 30-month-old C57BL/6NJ mice in 10 seconds (30-gauge needle). Mice were then subjected to behavioral testing 1 month after injection.

### Mouse fear-conditioning test.

Fear conditioning was performed as previously described to test changes in the hippocampus-involved memory of mice (94). Mice were allowed to acclimate to the testing environment at least 30 minutes before tests. Mice were placed in randomized groups to which the experimenter was blinded during the tests. Experiments were performed using the fear-conditioning system from Maze Engineers. On day 1, mice were placed in the fear-conditioning cage for 3 minutes to record pretraining freezing conditions. On day 2, mice received a training of an 18-second tone (2,000 Hz, 75 dB) followed by a 2-second electric shock (0.5 mA). On day 3, mice were again placed in the fear-conditioning cage for 3 minutes to record their after-training freezing conditions. Data were recorded and analyzed using ANY-maze software.

### Metabolic caging.

Mouse food and water intake were measured using a metabolic cage. Mice were randomly housed singly in metabolic cages with plastic walls and a wire mesh floor for 24 hours. Food and water intake were quantified by measuring food container weight and water volume before and after mouse housing.

### Morris water maze.

Mouse spatial learning and memory were examined using a Morris water maze. Mice were placed in randomized groups to which the experimenter was blinded during the tests. Mice were allowed to acclimate to the testing environment at least 0.5 hours before tests. Nontoxic tempera paint was added to an open swimming tank (200 cm diameter) filled with 2°C water to hide the platform (20 cm diameter, 1 cm below the water). The mice were trained to find the hidden platform in the tank. Four trials were performed each day for 12 days. Each trial started at a different position (northwest [NW], north [N], east [E], southeast [SE] quadrants), while the platform was kept in a single location (SW quadrant). Each trial lasted 60 seconds, followed by 30 seconds during which mice were allowed to remain on the platform to memorize the location of the platform. After 12 days of training, mice were subjected to a probe test in which the platform was removed. The latency they needed to reach the platform and the number of times they passed the previous platform location was analyzed using ANY-maze software to present mice learning curves and probe results, respectively.

### NOR.

Mouse learning and recognition memory was tested using NOR. Mice were placed in randomized groups to which the experimenter was blinded during the tests. Mice were kept in the empty arena for 10 minutes each day for 5 days during habituation. On training day, mice were exposed to the familiar arena with 2 identical objects placed at an equal distance. The next day, one of the objects was replaced with a novel object of similar height and volume but different shape and appearance as the familiar object to test mouse long-term recognition memory. Mice were allowed to explore objects for 10 minutes on both training and testing days. The time spent exploring each object was recorded and analyzed using ANY-maze software.

### Statistics.

Statistical analyses were performed using GraphPad Prism 8 software. Unpaired 2-way Student’s *t* test or 1- or 2-way ANOVA followed by Bonferroni post hoc analysis were applied in data analysis. Correlation analyses were performed using Pearson’s correlation coefficient or multiple linear regression. The Michaelis-Menten equation was used in evaluating drug effects. The data collected from mouse studies were presented as box-and-whisker plots displaying the median as a line within the box, interquartile range (IQR) as the box, and 95% CI as bars flanking the box. Within the main text, results are expressed as mean ± SEM. The data collected from participants were expressed as mean ± SD. Significance was concluded when the *P* value was less than 0.05.

### Study approval.

All animal studies are approved by the IACUC of the University of Kansas (protocol no. 272-01) and the NIH. Informed consent was collected from all participants, and the study adhered to the Declaration of Helsinki principles.

## Author contributions

JT, LG, TW, and KJ carried out experiments and collected the data. JT, LG, and HD performed the statistical analyses. JMZ provided GHSR-null mice. RHS provided postmortem human brain samples. LG, JMZ, RHS, and HD contributed to the design of experiments and helped with a critical reading of the manuscript. HD conceived the project, supervised the experiments, and wrote the manuscript.

## Supplementary Material

Supplemental data

## Version 1 05/22/2023

Electronic publication
