# Supplementary material for: Liver-expressed antimicrobial peptide 2 elevation contributes to age-associated cognitive decline
Source: JCI Insight. 2023 May 22;8(10):e166175. doi: 10.1172/jci.insight.166175 (PMC10322694; doi:10.1172/jci.insight.166175)
Supplement: Supplemental data [file jciinsight-8-166175-s150.pdf]

Supplementary figures and table:

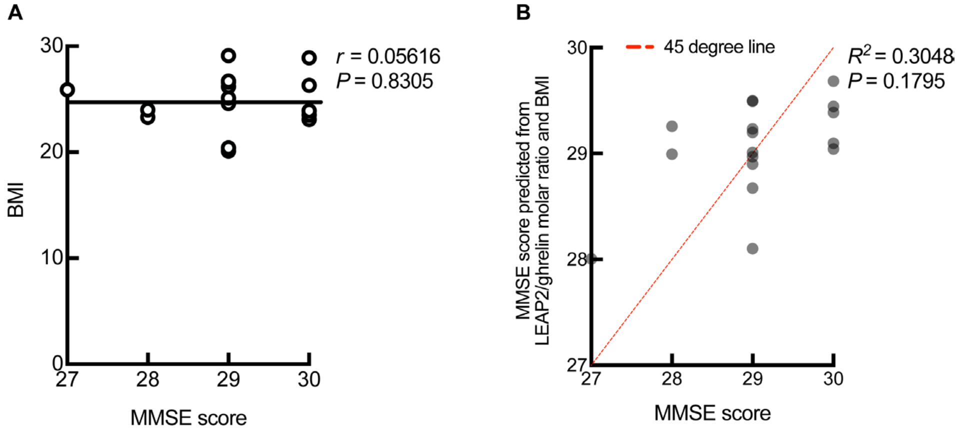

Supplementary Figure 1. Correlation analysis of MMSE scores and BMI in the tested elderly. **(A)** Correlation between MMSE scores and BMI was calculated using two-tailed Pearson correlation coefficients. *n* = 19 subjects. **(B)** Multiple linear regression of MMSE score prediction with LEAP2/ghrelin molar ratio and BMI as independent factors. Actual value (X axis) vs predicted value (Y axis). *n* = 19 subjects.

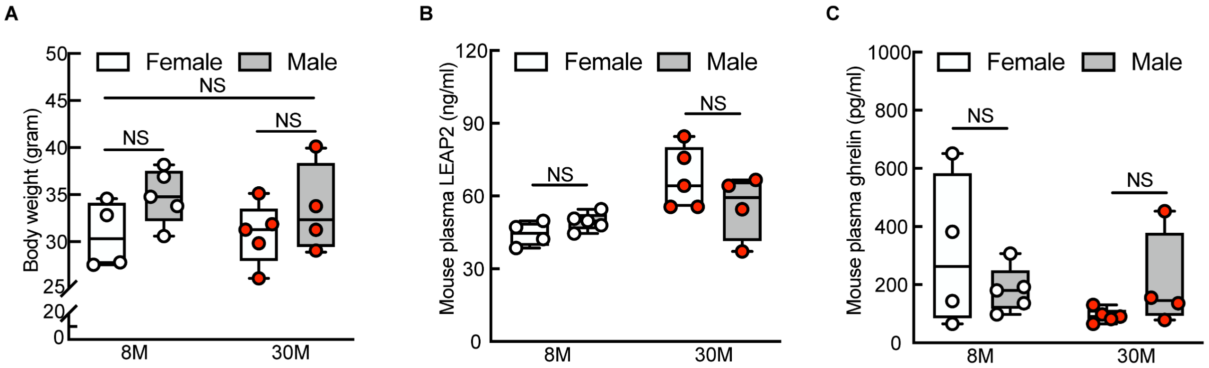

Supplementary Figure 2. Body weight measurement and sex effect examination in middle-aged and old mice. **(A)** The body weight (in grams) of 8-month-old and 30-month-old mice shows no sex difference. *n* = 4 female (8 months old), 5 male (8 months old), 5 female (30 months old), 4 male (30 months old). Two-way ANOVA followed by Bonferroni post hoc analysis. NS = not significant. All error bars represent means ± SEM. 8M = 8 months old mice, 30M = 30 months old mice. **(B)** Plasma LEAP2 or **(C)** plasma ghrelin shows no sex difference. *n* = 4 female (8 months old), 5 male (8 months old), 5 female (30 months old), 4 male (30 months old). Unpaired Student's t test. NS = not significant. 8M = 8 months old mice, 30M = 30 months old mice.

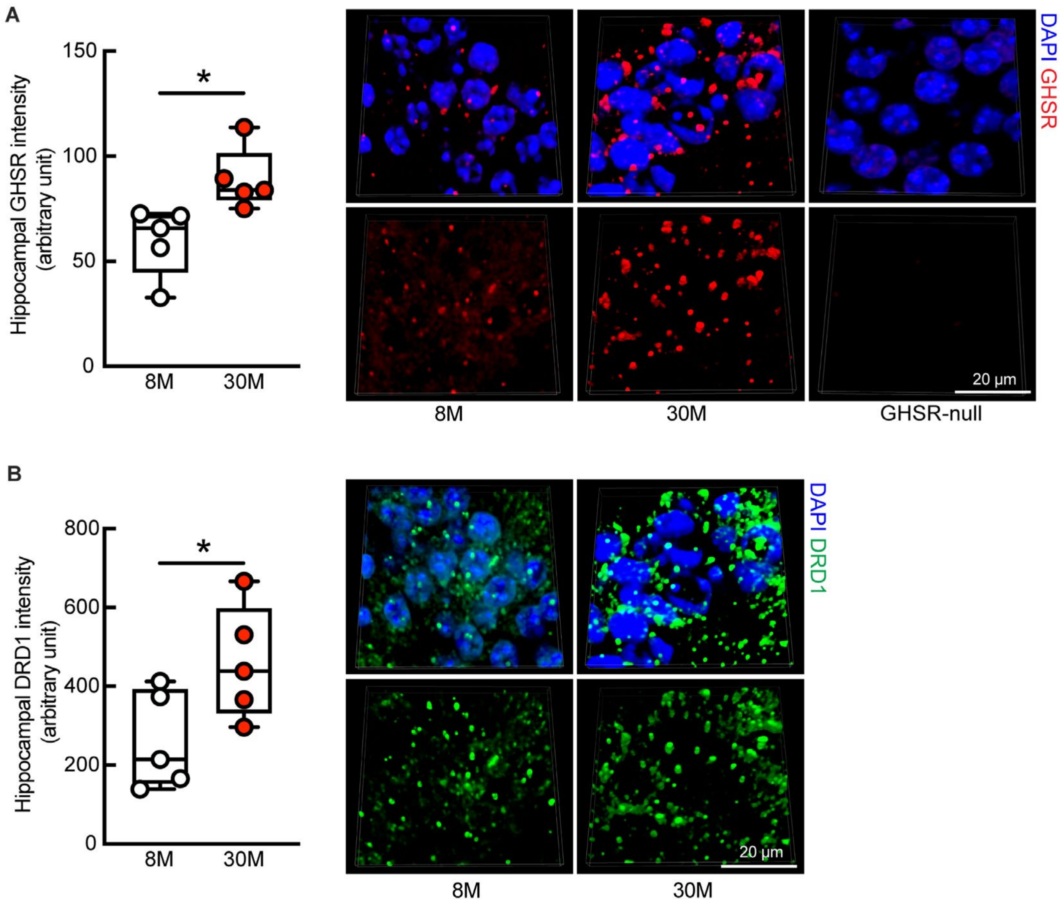

Supplementary Figure 3. Age-related expression changes of hippocampal GHSR and DRD1. **(A)** Upregulated hippocampal GHSR and DRD1 in 30-month-old mice. Immunostaining of hippocampal GHSR **(A)** and DRD1 **(B)**. Right panels are representative images. DAPI was used to label nucleus. GHSR-null mice were used as negative control for GHSR staining. n = 5 mice (8M and 30M), n = 2 GHSR-null mice. Unpaired Student's t test. *P < 0.05. Scale bar = 10 μm. 8M = 8 months old mice, 30M = 30 months old mice, GHSR-null = 8 months old GHSR-null mice.

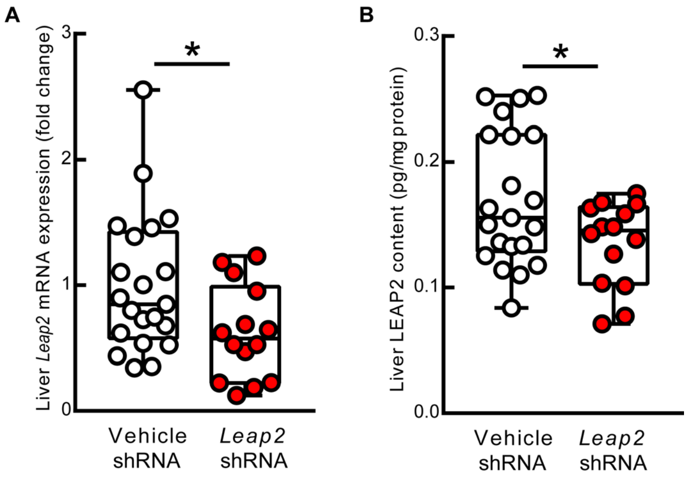

Supplementary Figure 4. Hepatic LEAP2 downregulation in LEAP2-manipulated old mice. Hepatic *Leap2* mRNA expression **(A)** and LEAP2 content **(B)** in vehicle- and *Leap2* shRNA-treated mice. $n = 21$ mice in vehicle shRNA group, 14 mice in *Leap2* shRNA group. Unpaired Student's t test. $*P < 0.05$.

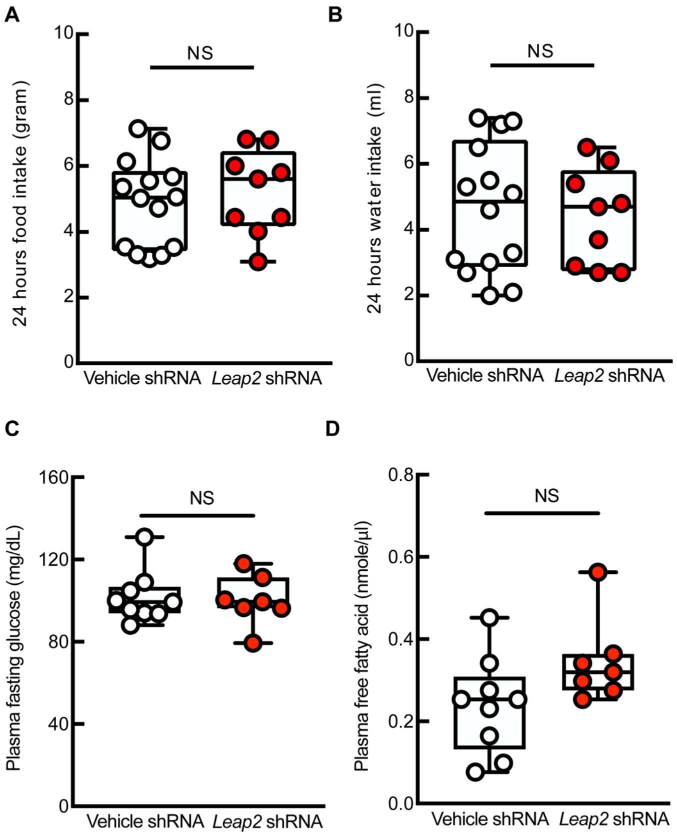

Supplementary Figure 5. No impact of LEAP2 downregulation on metabolism in aged mice. (A) 24-hour food intake in vehicle- and *Leap2* shRNA-treated mice. *n* = 14 mice in vehicle shRNA group, 9 mice in *Leap2* shRNA group. **(B)** 24-hour water intake in vehicle- and *Leap2* shRNA-treated mice. *n* = 14 mice in vehicle shRNA group, 9 mice in *Leap2* shRNA group. **(C)** Plasma fasting glucose in vehicle- and *Leap2* shRNA-treated mice. *n* = 9 mice in vehicle shRNA group, 7 mice in *Leap2* shRNA group. **(D)** Plasma free fatty acid in vehicle- and *Leap2* shRNA-treated mice. *n* = 9 mice in vehicle shRNA group, 7 mice in *Leap2* shRNA group. Statistical analysis: Unpaired Student's *t* test. NS = not significant.

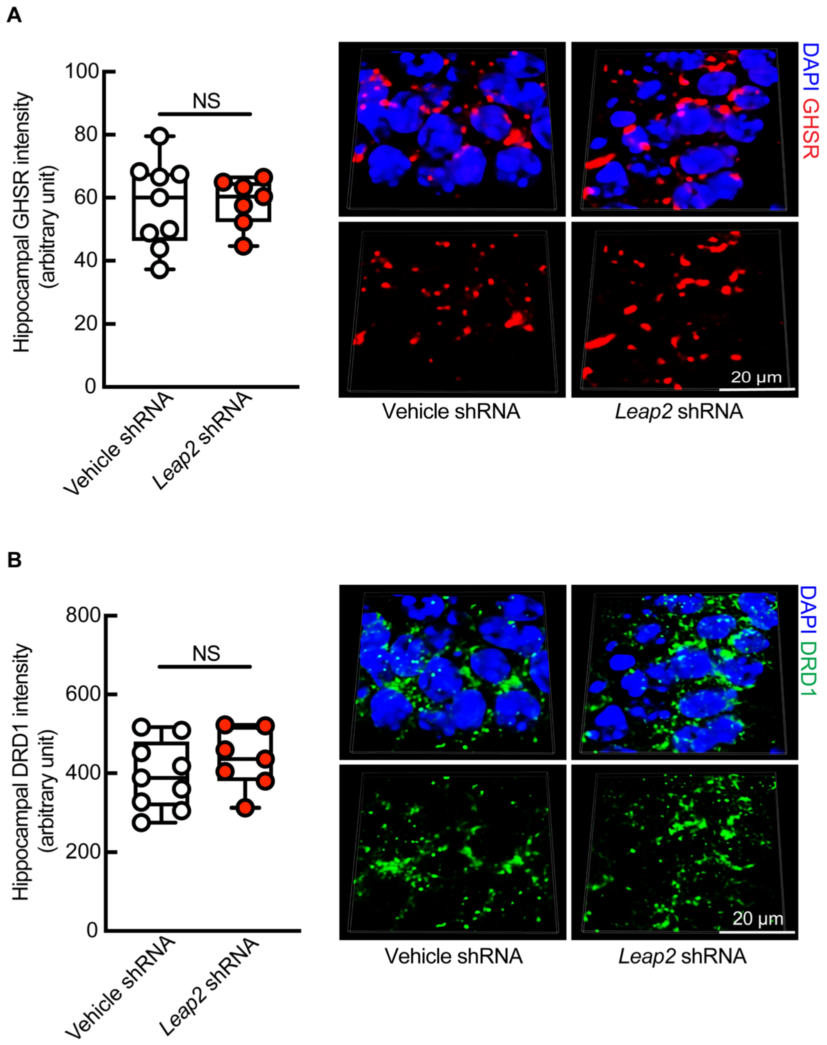

Supplementary Figure 6. No influence of LEAP2 downregulation on hippocampal GHSR and DRD1 expression in aged mice. (A) Hippocampal GHSR staining in vehicle and *Leap2* shRNA-treated mice. *n* = 9 mice in vehicle shRNA group, 7 mice in *Leap2* shRNA group. Unpaired Student's t test. NS = not significant. Right panels are the representative images. Scale bar = 10 μm. **(B)** Hippocampal DRD1 staining in vehicle and *Leap2* shRNA-treated mice. *n* = 9 mice in vehicle shRNA group,7 mice in *Leap2* shRNA group. Unpaired Student's t test. NS = not significant. Right panels are the representative images. Scale bar = 10 μm.

sTable 1. Demographic characteristics of human subjects.

Characteristics	Value ± SD
Age (years)	71.68 ± 7.432
Sex (male/female)	7/12
Race	
White	64%
African American	36%
Education (years)	16.43 ± 1.989
Height (inches)	65.66 ± 3.368
Weight (lbs)	153.1 ± 23.56
BMI	24.87 ± 2.725
MMSE score	29.06 ± 0.8269

sTable 1. Demographic information of the cohort including age, sex, race, education, height, weight, BMI, and MMSE score are evaluated. n = 19 subjects.
